# The medial dorsal thalamic nucleus and the medial prefrontal cortex of the rat function together to support associative recognition and recency but not item recognition

**DOI:** 10.1101/lm.028266.112

**Published:** 2013-01

**Authors:** Laura Cross, Malcolm W. Brown, John P. Aggleton, E. Clea Warburton

**Affiliations:** 1 School of Physiology and Pharmacology, University of Bristol, Bristol, Avon BS8 1TD, United Kingdom; 2 School of Psychology, Cardiff University, Cardiff CA10 3AT, United Kingdom

## Abstract

In humans recognition memory deficits, a typical feature of diencephalic amnesia, have been tentatively linked to mediodorsal thalamic nucleus (MD) damage. Animal studies have occasionally investigated the role of the MD in single-item recognition, but have not systematically analyzed its involvement in other recognition memory processes. In Experiment 1 rats with bilateral excitotoxic lesions in the MD or the medial prefrontal cortex (mPFC) were tested in tasks that assessed single-item recognition (novel object preference), associative recognition memory (object-in-place), and recency discrimination (recency memory task). Experiment 2 examined the functional importance of the interactions between the MD and mPFC using disconnection techniques. Unilateral excitotoxic lesions were placed in both the MD and the mPFC in either the same (MD + mPFC Ipsi) or opposite hemispheres (MD + mPFC Contra group). Bilateral lesions in the MD or mPFC impaired object-in-place and recency memory tasks, but had no effect on novel object preference. In Experiment 2 the MD + mPFC Contra group was significantly impaired in the object-in-place and recency memory tasks compared with the MD + mPFC Ipsi group, but novel object preference was intact. Thus, connections between the MD and mPFC are critical for recognition memory when the discriminations involve associative or recency information. However, the rodent MD is not necessary for single-item recognition memory.

Recognition memory, the ability to judge the prior occurrence of a stimulus, involves multiple processes and requires multiple brain regions. Research into human amnesia shows that the medial diencephalon is critical for normal recognition memory, although neuropsychological studies have largely failed to link the contributions of particular diencephalic sites to aspects of recognition (Aggleton et al. 2011). The principal reason is that the pathology in human clinical cases almost invariably involves multiple diencephalic nuclei.

One diencephalic site potentially linked to human recognition memory is the mediodorsal thalamic nucleus (MD) (Victor et al. 1971; Victor 1987; Parkin et al. 1994; Isaac et al. 1998). This link is more evident in animal lesion experiments with monkeys, which have consistently indicated a role for the MD in item recognition (Aggleton and Mishkin 1983b; Zola-Morgan and Squire 1985; Parker et al. 1997). From such findings, Aggleton and Brown (1999) proposed a model of item recognition memory that incorporates the MD within a larger neural network centered on the perirhinal cortex and involving the prefrontal cortex (Aggleton and Brown 1999). This model is supported by the anatomical connections from the perirhinal cortex to the MD (Russchen et al. 1987; Saunders et al. 2005) and the observation that the primate perirhinal cortex, medial prefrontal cortex (mPFC), and MD contain neuronal populations that signal information concerning prior stimulus occurrence (Fahy et al. 1993; Xiang and Brown 1998, 2004).

The importance of the rat MD for single-item recognition is, however, far less clear-cut (Mumby et al. 1993; Parker et al. 1997). This uncertainty may reflect differences in the learning demands of the various studies (Aggleton et al. 2011). The first goal was, therefore, to reexamine the importance of the rat MD for item recognition using spontaneous tasks that minimize procedural learning. The second goal was to extend the classes of recognition problem used to examine the rat MD. In addition to single-item recognition (tested using the novel object recognition task [Fig. 1A]), the present study examined object spatial memory (Fig. 1B) associative recognition (distinguishing between multiple familiar items in their original or novel configuration assessed using an object-in-place task [Fig. 1C]) and recency discriminations (assessed using a recency recognition task [Fig. 1D]). The choice of task was prompted by the finding that mPFC lesions in rats spare single-item recognition, but disrupt associative recognition and recency judgments (Barker et al. 2007). Furthermore, in view of the dense, reciprocal interconnections between the MD and mPFC (Krettek and Price 1977; Groenewegen 1988; Ray and Price 1992, 1993; Taber et al. 2004) it might be predicted that the MD functions in concert with the prefrontal cortex to enable associative recognition and recency judgments selectively. This prediction is supported indirectly by some of the parallel effects of MD lesions and prefrontal lesions on memory in humans (Milner and Petrides 1984; Sandson et al. 1991; Van der Werf et al. 2000), monkeys (Parker and Gaffan 1998), and rats (Hunt and Aggleton 1998; Dias and Aggleton 2000), but remains to be tested directly. Thus, the third goal of the present study was to use disconnection procedures that examine the interaction of MD and mPFC.

**Figure 1.**
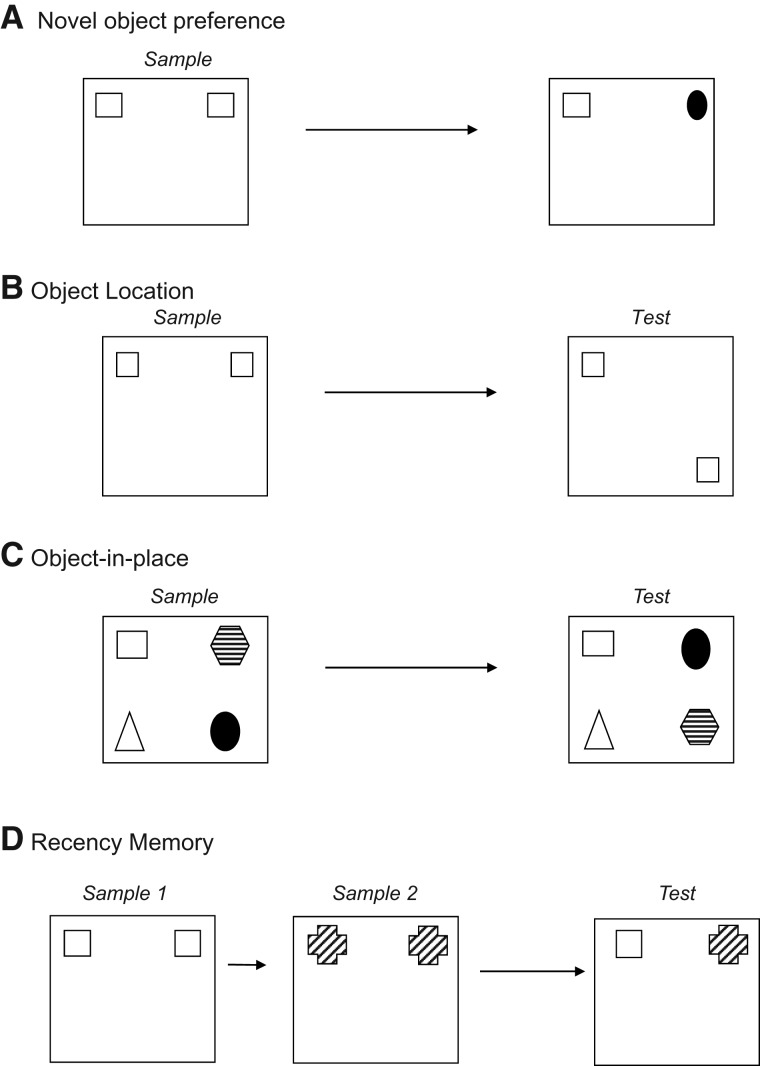
Diagram of the four object recognition memory tasks. (*A*) Novel object recognition task. (*B*) Object location task. (*C*) Object-in-place task. (*D*) Recency task.

## Results

### Histology

#### Experiment 1: Bilateral mPFC lesions

**Figure 2.**
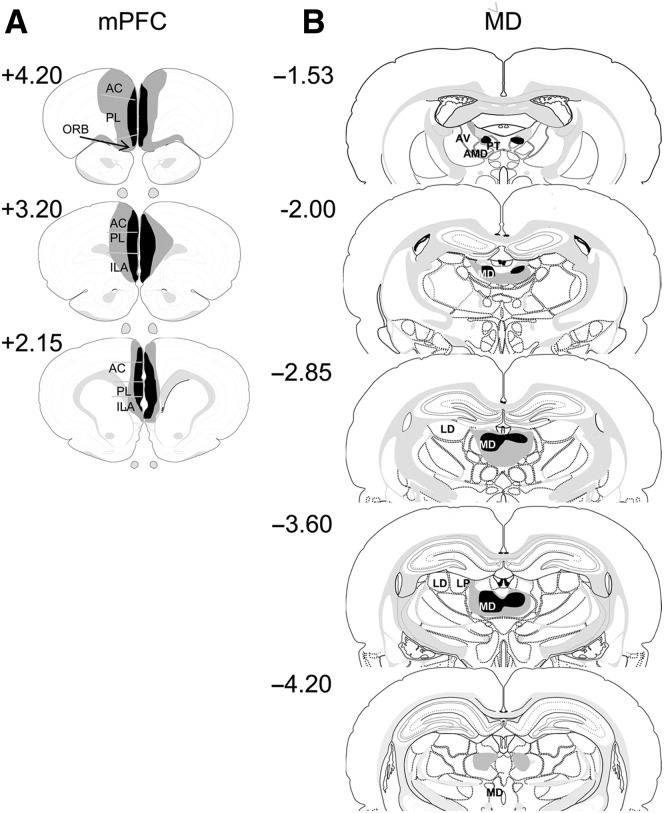
Diagrammatic reconstructions showing the cases with the largest (gray) and smallest (black) lesions in the mPFC (*A*) and MD (*B*) lesion groups. The numbers correspond to the approximate position relative to bregma (Swanson 1998). (AC) Anterior cingulate cortex; (AV) anteroventral thalamic nucleus; (AMD) anteromedial thalamic nucleus; (ILA) infralimbic cortex; (LD) lateral dorsal thalamic nucleus; (LP) lateral posterior thalamic nucleus; (MD) mediodorsal thalamic nucleus; (ORB) orbital frontal cortex; (PL) prelimbic cortex; (PT) parataenial nucleus.

All the animals had bilateral damage in the mPFC. All but two animals had complete lesions of both the prelimbic cortex (PL) and the infralimbic (IL) cortex. The two other animals had sparing in the deep layers. All the animals also had minor cingulate cortex damage around the border with the PL. Three animals had a small amount of damage to the medial orbital cortex and ventral orbital cortex. Two animals had lesions that extended to a minor degree into the secondary motor cortex, and one animal had sustained damage in the dorsal peduncular cortex. Diagrammatic representations of the cases with the largest and smallest lesions are shown in Figure 2A.

#### Experiment 1: Bilateral MD lesions

Four animals were excluded, three due to incomplete damage to the MD, particularly in one hemisphere, and one as there was additional bilateral damage to anterior thalamic nuclei with some sparing of the MD. In the remaining eight cases there was bilateral damage to the MD; however, all showed a minor amount (<10%) of sparing in the lateral extremes of one hemisphere. All the animals had some unilateral damage to the central lateral nucleus and partial damage to the central medial nucleus. For six of the animals, minor damage (<10%) was incurred unilaterally in the ventral anterior lateral nucleus and anterodorsal nucleus. Four animals had extensive damage to the paraventricular nucleus and five had bilateral damage to the posterior parataenial nucleus. Three animals showed minor unilateral (<10%) damage to the dentate gyrus (DG) and one animal sustained minor damage to the DG bilaterally. Importantly, the MD was the only common site of tissue loss across the cases. Diagrammatic representations of the cases with the largest and smallest lesions are shown in Figure 2B.

#### Experiment 2: Ipsilateral MD + mPFC lesions

**Figure 3.**
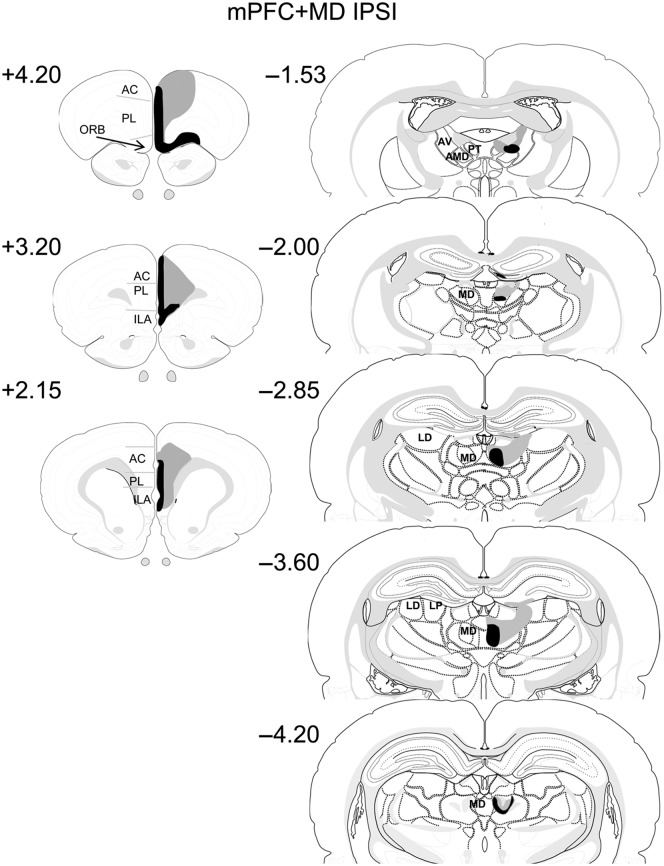
Diagrammatic reconstructions showing the cases with the largest (gray) and smallest (black) lesions in the mPFC-MD Ipsi lesion group. The numbers correspond to the approximate position relative to bregma (Swanson 1998). (AC) anterior cingulate cortex; (AV) anteroventral thalamic nucleus; (AMD) anteromedial thalamic nucleus; (ILA) infralimbic cortex; (LD) lateral dorsal thalamic nucleus; (LP) lateral posterior thalamic nucleus; (MD) mediodorsal thalamic nucleus; (ORB) orbital frontal cortex; (PL) prelimbic cortex; (PT) parataenial nucleus.

Two animals were excluded as there was no damage in the MD. In the remaining 10 cases all the animals had near-complete unilateral lesions in the MD and mPFC. Thus within the mPFC there was extensive damage to the prelimbic and the infralimbic cortices, although one animal showed posterior and deep layer sparing of the PL. In one case the primary motor cortex showed minor damage (<10%) and in another there was evidence of very minor damage to the corpus callosum. In half the cases there was minor damage in the cingulate cortex, although this damage was confined to the border of the PL. All the animals had extensive unilateral damage in the MD. One animal had some minor sparing in the lateral MD, while two had additional lateral damage affecting the dorsomedial part of the lateral dorsal thalamus; in one case this damage extended into the ventrolateral part of the lateral dorsal thalamus and the medial rostral part of the lateral posterior thalamus (largest lesion in Figure 3). Seven animals showed a small amount of unilateral damage in the DG and anterior dorsal thalamus at the site of the MD injection. Diagrammatic representations of the cases with the largest and smallest lesions are shown in Figure 3.

#### Contralateral MD + mPFC lesions

**Figure 4.**
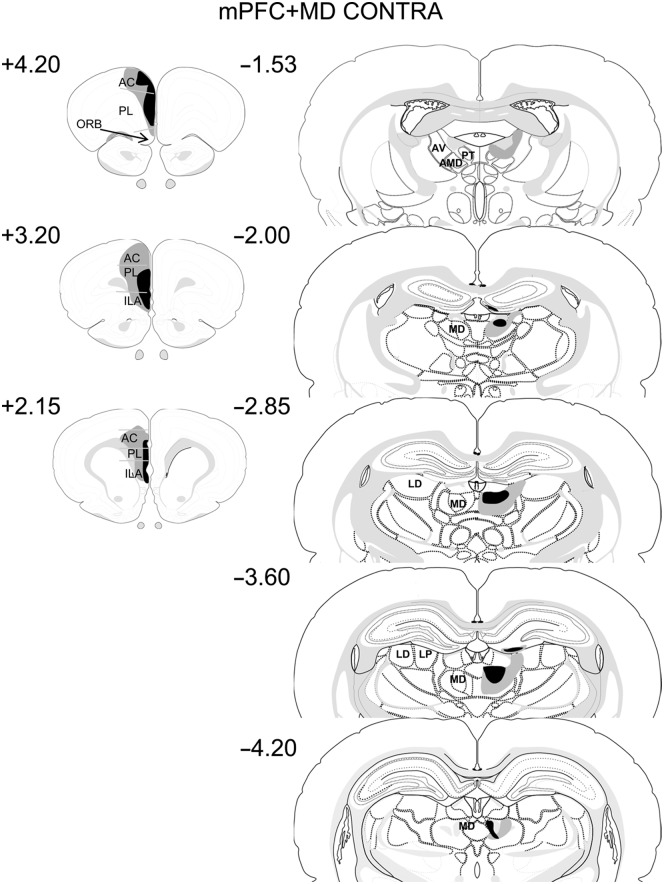
Diagrammatic reconstructions showing the cases with the largest (gray) and smallest (black) lesions in the mPFC–MD Contra lesion group. The numbers correspond to the approximate position relative to bregma (Swanson 1998). (AC) anterior cingulate cortex; (AV) anteroventral thalamic nucleus; (AMD) anteromedial thalamic nucleus; (ILA) infralimbic cortex; (LD) lateral dorsal thalamic nucleus; (LP) lateral posterior thalamic nucleus; (MD) mediodorsal thalamic nucleus; (ORB) orbital frontal cortex; (PL) prelimbic cortex; (PT) parataenial nucleus.

Two animals were excluded. One animal was excluded as there was no damage in the MD and one because of extra damage in the mammillothalamic tract region. In the remaining 10 cases all the animals had unilateral lesions in the MD and mPFC in the contralateral hemispheres. All the animals had large unilateral lesions in the prelimbic and infralimbic cortices, although one animal showed sparing in the deep layers of the PL. Two animals showed minimal (<10%) damage to the motor cortex. In all cases, the cingulate cortex was damaged by about 50% in the region closest to the PL. In all cases there was a large unilateral lesion in the MD. The animals showed a small degree of sparing of either the anterior or posterior parts of the MD, but never of both. Six animals also showed a degree of unilateral damage to the DG and the anterior dorsal thalamic nucleus around the area of the injection site. Six animals also had minor (<10%) damage to the intramedullary lamina. Diagrammatic representations of the cases with the largest and smallest lesions are shown in Figure 4.

### Behavior: Experiment 1

After histological analysis, the final group numbers were as follows: sham, *n* = 12; mPFC, *n* = 12; MD, *n* = 8. As stated, those animals that did not complete the necessary levels of exploration were excluded from the final analyses, as indicated by reduced degrees of freedom in the quoted statistical tests.

#### Novel object recognition task

##### Recognition during the test phase

**Figure 5.**
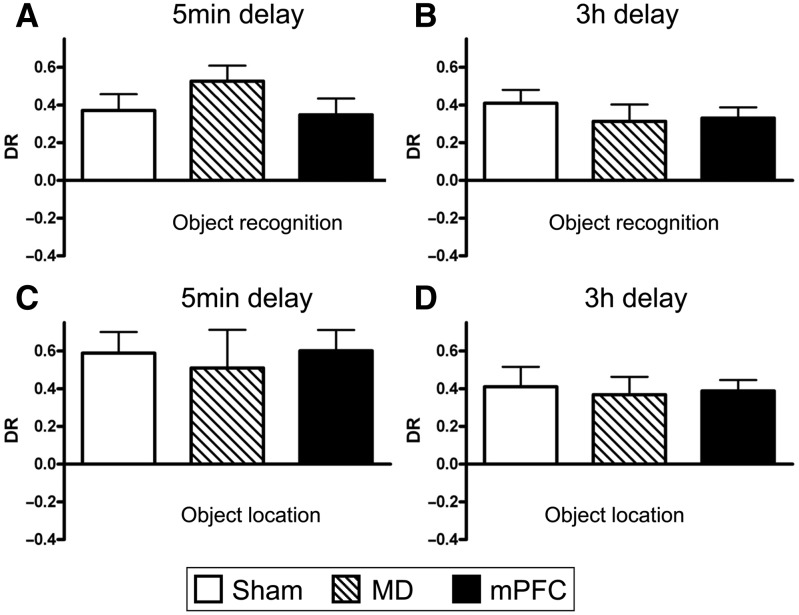
Performance of the bilateral MD and mPFC lesion groups in object recognition and object location tasks when tested with a 5-min or 3-h delay. (*A*) Object recognition performance at a 5-min delay. (*B*) Object recognition performance at a 3-h delay. (*C*) Object location performance at a 5-min delay. (*D*) Object location performance at a 3-h delay. Shown for each group is the mean (±SEM) discrimination ratio (DR).

Figure 5, A and B, shows the performance of all three groups (MD, mPFC, and sham) in the test phase following retention delays of either 5 min (Fig. 5A) or 3 h (Fig. 5B). Two-way ANOVA with the lesion and delay as factors showed that there was no significant main effect of the lesion (*F*_(2,57)_ = 0.5, *P* > 0.1), no significant effect of the delay (*F*_(1,57)_ = 0.9, *P* > 0.1), and no significant lesion-by-delay interaction (*F*_(2,57)_ = 1.8, *P* > 0.1). Further analyses revealed that all groups showed significant discrimination between novel and familiar objects across both delays (5-min sham: *t*_(11)_ = 4.2, *P* < 0.001; MD: *t*_(7)_ = 6.3, *P* < 0.001; mPFC: *t*_(11)_ = 4.1, *P* < 0.01; 3-h sham: *t*_(11)_ = 5.7, *P* < 0.001; MD: *t*_(7)_ = 3.5, *P* < 0.001; mPFC: *t*_(11)_ = 5.8, *P* < 0.001).

These data indicate that bilateral lesions in neither the MD nor the mPFC affect object recognition memory.

##### Exploration in sample and test phases

Analysis of the total amount of exploration completed in the sample phase or test phase revealed no effect of the lesion in either phase at either delay (all *F*’s < 2.0). The data are presented in Table 1. In addition, there was no difference in the amount of exploration completed in the test phase following a 5-min or 3-h delay *F*_(1,28)_ = 3.64, *P* > 0.05.

**Table 1.**
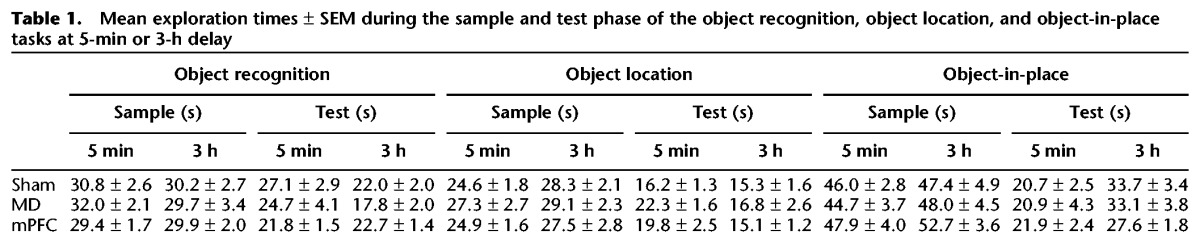
Mean exploration times ± SEM during the sample and test phase of the object recognition, object location, and object-in-place tasks at 5-min or 3-h delay

#### Object location task

##### Recognition during the test phase

The performance of all the groups in the object location task is shown in Figure 5, C and D. At the 5-min delay experiment one animal from each lesion group was excluded due to insufficient levels of object exploration, and at the 3-h delay two MD animals were excluded. ANOVA with the lesion group and delay as factors revealed no significant main effect of the lesion (*F*_(2,51)_ = 0.1, *P* > 0.1) and of the delay (*F*_(1,51)_ = 3.4, *P* > 0.1), and no significant lesion-by-delay interaction (*F*_(2,51)_ = 0.04, *P* > 0.1). Further analysis showed that all the lesion groups were able to discriminate between novel and familiar locations following a delay of 5 min (sham: *t*_(10)_ = 5.2, *P* < 0.001; MD: *t*_(6)_ = 2.5, *P* < 0.05; mPFC: *t*_(10)_ = 5.5, *P* < 0.001) or 3 h (sham: *t*_(10)_ = 3.9, *P* < 0.01; MD: *t*_(5)_ = 3.9, *P* < 0.01; mPFC: *t*_(10)_ = 6.7, *P* < 0.001).

These data indicate that the bilateral lesions in the MD and mPFC had no effect on the performance of the object location task.

##### Exploration in sample and test phases

Analysis of the total amount of exploration completed in the sample phase or test phase revealed no effect of the lesion (all *F*’s < 1.5). The data are presented in Table 1.

#### Object-in-place task

##### Recognition during the test phase

**Figure 6.**
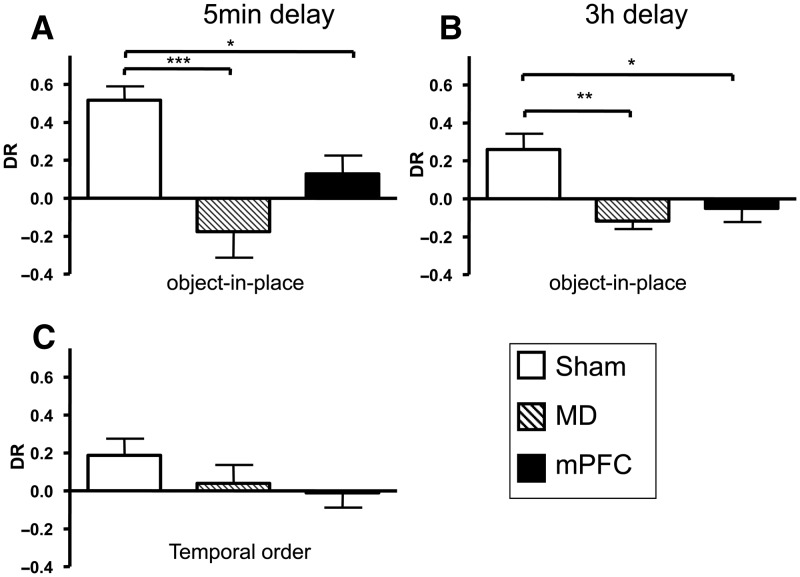
Performance of bilateral MD and mPFC lesion groups in the object-in-place and temporal order memory tasks. (*A*) Object-in-place performance following a 5-min delay. (*B*) Object-in-place performance following a 3-h delay. (*C*) Temporal order memory performance. (*) *P* < 0.05, (**) *P* < 0.01, (***) *P* < 0.001. Shown for each group is the mean (±SEM) discrimination ratio (DR).

Figure 6, A and B, shows the performance of the bilateral MD and mPFC groups in the object-in-place task following a 5-min (Fig. 6A) or 3-h delay (Fig. 6B). One mPFC was excluded from the analysis at the 5-min delay. Two-way ANOVA with the lesion group and delay as factors revealed a significant main effect of the lesion (*F*_(2,57)_ = 19.3, *P* < 0.001), but no effect of the delay (*F*_(1,57)_ = 3.1, *P* > 0.1) and no significant lesion-by-delay interaction (*F*_(2,57)_ = 1.5, *P* > 0.1). Post hoc analyses showed that the performances of the MD and mPFC groups were significantly worse than that of the sham group following both a 5-min delay (MD, *P* < 0.001; mPFC, *P* < 0.05) and a 3-h delay (MD, *P* < 0.01; mPFC, *P* < 0.05). The sham control group displayed significant discrimination between the stationary objects and the objects that had exchanged position following both retention delays (5 min: *t*_(11)_ = 7.1, *P* < 0.001; 3 h: *t*_(11)_= 3.1, *P* < 0.01), but the mPFC group did not (5 min: mPFC: *t*_(10)_ = 1.3, *P* > 0.1; 3 h: mPFC: *t*_(11)_ = 0.7, *P* > 0.1). The MD group also did not discriminate at the 5-min delay (MD: *t*_(7)_ = 1.3, *P* > 0.1), but at the 3-h delay this group showed a significant preference for the stationary objects compared with the objects that had exchanged position (MD: *t*_(7)_ = −2.7, *P* < 0.05); hence the MD lesion produced only partial impairment at the 3-h delay.

These data suggest that both the MD and mPFC are necessary for normal object-in-place associative memory.

##### Exploration in sample and test phases

Analysis of the total amount of exploration conducted in the sample phase or test phase revealed no effect of the lesion following either delay (all *F*’s < 1.5). The exploration data are shown in Table 1.

#### Recency memory task

##### Recognition during the test phase

The performances of the sham, bilateral MD, and mPFC lesion groups in the recency memory task are shown in Figure 6C, and a statistical comparison of the mean discrimination ratios against zero performance showed that only the sham control group significantly discriminated between the ‘old’ and ‘recent’ objects (sham: *t*_(11)_ = 2.5, *P* < 0.05), while the MD and mPFC groups did not (MD: *t*_(7)_ = 0.5, *P* > 0.1; mPFC: *t*_(11)_ = 0.2, *P* > 0.1). Thus, while the ANOVA did not reveal any significant group effect (*F*_(2,29)_ = 1.0, *P* > 0.1), both lesion groups failed to show an ability to use recency information to effect a recognition memory judgment.

##### Exploration in sample and test phases

Analysis of the total amount of exploration conducted in the sample phases or test phase revealed no effect of the lesion (all *F*’s < 1.5). The data are shown in Table 2.

**Table 2.**
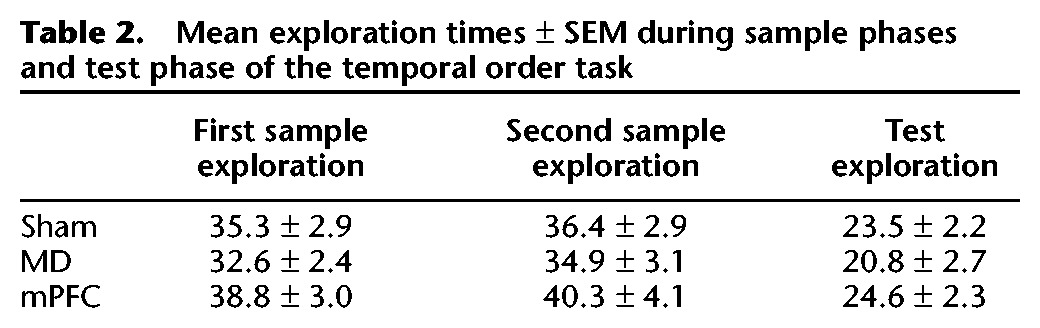
Mean exploration times ± SEM during sample phases and test phase of the temporal order task

### Behavior: Experiment 2

Following histological analysis the final numbers of animals in the MD + mPFC Ipsi and MD + mPFC Contra lesion groups were 10 in each group. As stated, animals that did not complete the necessary levels of exploration were excluded from the final analyses, as indicated by reduced degrees of freedom in the quoted statistical tests.

#### Novel object recognition task

##### Recognition during the test phase

**Figure 7.**
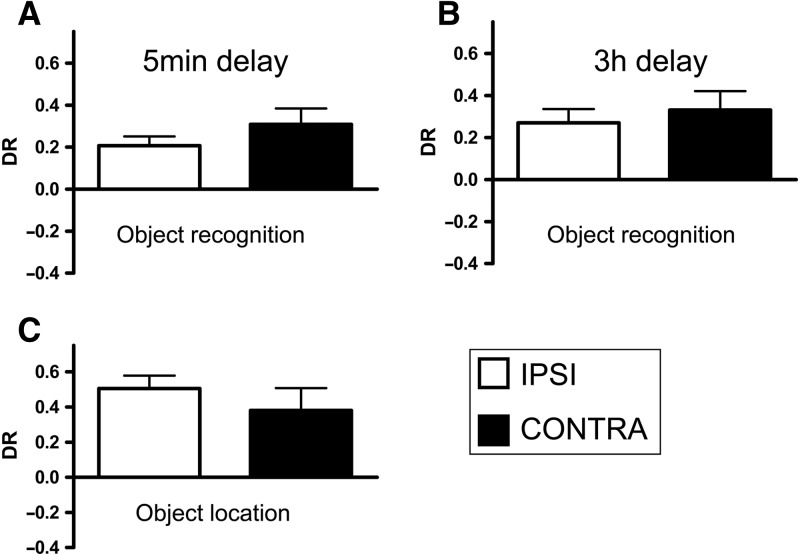
Performance of the MD + mPFC Ipsi and MD + mPFC Contra lesion groups in object recognition and object location tasks following a 5-min delay or 3-h delay. (*A*) Object recognition performance following a 5-min delay. (*B*) Object recognition performance following a 3-h delay. (*C*) Object location performance following a 5-min delay. Shown for each group is the mean (±SEM) discrimination ratio (DR).

Figure 7, A and B, shows the performance of the MD + mPFC Ipsi and MD + mPFC Contra lesion groups in the object recognition task and shows that the performance of these two groups did not differ from one another. ANOVA with the lesion group and delay as factors showed no significant main effect of lesion type (*F*_(1,36)_= 1.3, *P* > 0.1), of delay (*F*_(1,36)_= 0.3, *P* > 0.1), and no lesion type-by-delay interaction (*F*_(1,36)_= 0.08, *P* > 0.1). Additional analyses revealed that both groups significantly discriminated the novel from the familiar objects following delays of 5 min (MD + mPFC Contra: *t*_(9)_ = 4.1, *P* < 0.01; MD + mPFC Ipsi: *t*_(9)_ = 4.7, *P* < 0.01), and 3 h (MD + mPFC Contra: *t*_(9)_ = 3.7, *P* < 0.01; MD + mPFC Ipsi: *t*_(9)_ = 4.1, *P* < 0.001).

##### Exploration in sample and test phases

Analysis of the total amount of exploration conducted in the sample phase or test phase of the novel object recognition task revealed no significant differences at any delay (all *F*’s < 2.0). The data are shown in Table 3.

**Table 3.**
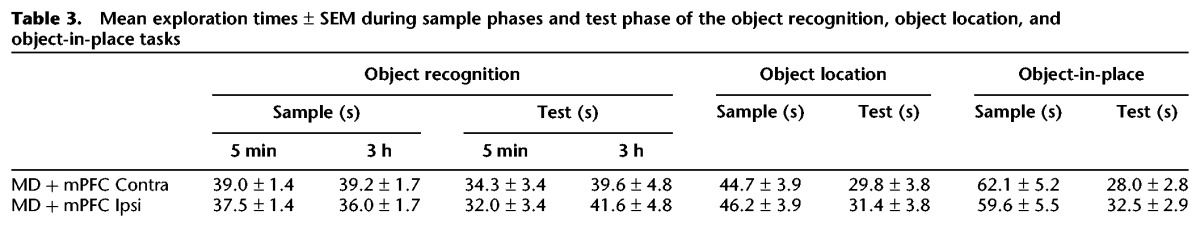
Mean exploration times ± SEM during sample phases and test phase of the object recognition, object location, and object-in-place tasks

#### Object location task

##### Recognition during the test phase

The performances of the MD + mPFC Ipsi and MD + mPFC Contra lesion groups in the object location task following a 3-h delay are shown in Figure 7C; the two lesion groups did not differ from one another. Analysis revealed no effect of lesion type (*F*_(1,18)_= 0.7, *P* > 0.1) and both groups showed significant discrimination between novel and familiar locations (MD + mPFC Ipsi: *t*_(9)_ = 6.8, *P* < 0.001; MD + mPFC Contra: *t*_(9)_ = 3.0, *P* < 0.01).

##### Exploration in sample and test phases

Analyses of the total amount of exploration in the sample or test phases of the object location task revealed no significant differences (all *F*’s < 1.0). The data are shown in Table 3.

#### Object-in-place task

##### Recognition during the test phase

**Figure 8.**
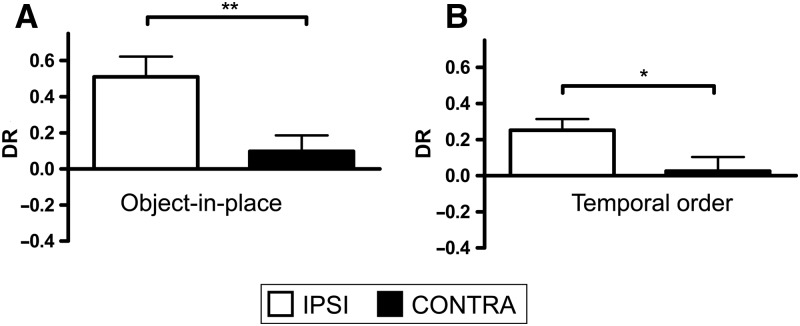
Performance of the MD + mPFC Ipsi and MD + mPFC Contra lesion groups in object-in-place and temporal order memory tasks. (*A*) Object-in-place task performance. (*B*) Temporal order memory performance task. (*) *P* < 0.05, (**) *P* < 0.01. Shown for each group is the mean (±SEM) discrimination ratio (DR).

Figure 8A shows the performances of the MD + mPFC Ipsi and MD + mPFC Contra lesion groups in the object-in-place task. One animal from the MD + mPFC group was excluded from the analyses due to insufficient levels of exploration. One-way ANOVA confirmed a significant main effect of lesion type (*F*_(1,18)_= 8.6, *P* < 0.01). Subsequent analyses confirmed that only the MD + mPFC Ipsi lesion group showed significant discrimination between the rearranged and unmoved objects in the test phase (MD + mPFC Ipsi: *t*_(8)_ = 4.6, *P* < 0.01; MD + mPFC Contra: *t*_(9)_ = 1.1, *P* > 0.1). Thus contralateral unilateral lesions in the MD and mPFC resulted in significant impairments in object-in-place performance.

##### Exploration in sample and test phases

Analysis of the total amount of exploration conducted in the sample or test phase of the object-in-place task revealed no significant differences (all *F*’s < 1.0). The data are shown in Table 3.

#### Recency task

##### Recognition during the test phase

Figure 8B shows the performance of both lesion groups in the recency task. One-way ANOVA showed a significant main effect of lesion type (*F*_(1,18)_ = 4.8, *P* < 0.05). Additional analyses revealed that only the MD + mPFC Ipsi lesion group showed a significantly greater preference for the object previously seen in the first sample phase (MD + mPFC Ipsi: *t*_(9)_ = 3.6, *P* < 0.01; MD + mPFC Contra: *t*_(9)_ = 0.7, *P* > 0.1). Contralateral unilateral lesions in the MD and mPFC significantly impaired performance in the recency task compared with animals with ipsilateral lesions.

##### Exploration in sample and test phases

Analysis of the total amounts of exploration conducted in the recency task revealed no effect of the lesion in either the sample phase or the test phase (all *F*’s < 1.0). The data are shown in Table 4.

**Table 4.**
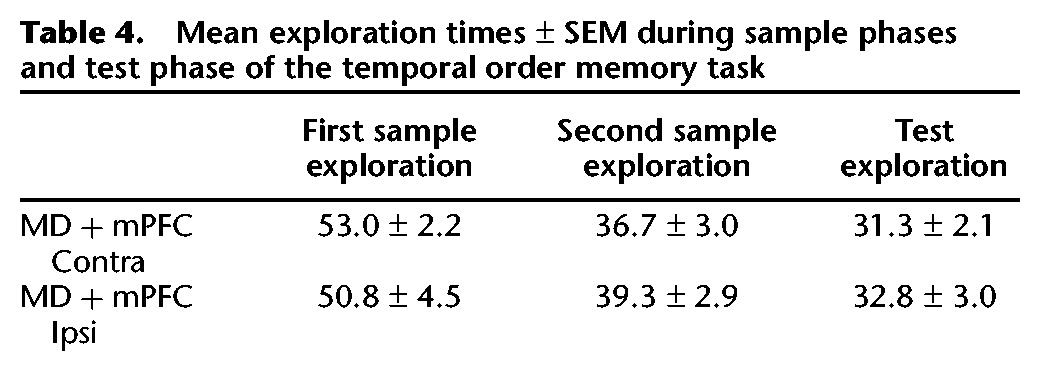
Mean exploration times ± SEM during sample phases and test phase of the temporal order memory task

## Discussion

The present study is the first to examine systematically the mnemonic importance of the rodent MD in recognition memory and it revealed four important findings. First, the MD was shown not to be necessary for single-item recognition. Second, the MD was found to be critical for object-in-place associative recognition and recency recognition. Third, this study clearly showed that the effects of MD damage on all the recognition memory tasks tested in the current study using these measurements were indistinguishable from the effects of damage to the mPFC. The fourth finding was that disconnection of the MD and mPFC significantly impaired object-in-place and recency recognition memory. Thus together these results demonstrate that the MD may be incorporated within a neural circuit for recognition memory when judgments of prior occurrence involve a spatial/associational or recency component.

MD damage has been shown to impair delayed nonmatching-to-sample (DNMS) tasks in primates (Aggleton and Mishkin 1983a; Zola-Morgan and Squire 1985); however, the results for rats appear to be less consistent (Aggleton et al. 2011). Tests using reinforced DNMS have found that MD lesions in rats impair the learning of task contingencies (Hunt and Aggleton 1991; Mumby et al. 1993), but recognition performance over relatively short delays appears normal, following task mastery. The single previous study to use spontaneous object recognition (Mitchell and Dalrymple-Alford 2005) reported no effect of MD lesions after the one (2-h) retention delay examined.

In light of the contradictory evidence concerning the importance of the MD in single-item recognition, the findings from the present study, i.e., that bilateral MD lesions have no effect on novel object recognition after a short or long retention delay are of importance as they suggest that the deficits previously seen in rats when using rewarded DNMS (Hunt and Aggleton 1991; Mumby et al. 1993) may reflect the effects of MD damage on aspects of task performance other than recognition. One possible candidate is reward association learning, a process critical for DNMS task acquisition, and indeed previous studies show that reward learning is compromised by MD damage (Corbit et al. 2003). Numerous studies have shown the perirhinal cortex to be critical for object recognition memory (Ennaceur et al. 1996; Ennaceur and Aggleton 1997; Bussey et al. 2000; Barker et al. 2007) and in light of the present null result it is noteworthy that anatomical studies have shown little evidence of direct connections between the MD and perirhinal cortex in the rat (Ray and Price 1992). This arrangement appears different to that in the rat brain where direct perirhinal projections to the MD have been traced (Aggleton and Mishkin 1983a; Russchen et al. 1987).

There are strong anatomical connections between the MD and the mPFC and, as our previous studies demonstrated the importance of the mPFC in certain recognition memory processes, it was important to compare directly the role of the MD with that of the mPFC in different forms of recognition memory other than single-item object recognition, i.e., object location recognition, object-in-place, and recency memory. Bilateral lesions in both the MD and mPFC significantly impaired object-in-place and recency memory performance, but had no apparent effect on object location memory. The object location task assesses the rat’s ability to recognize that an object is in a previously unoccupied place, but does not require identification of which object goes where, and therefore does not require associative recognition. While regions important for spatial learning, e.g., the hippocampus and fornix, are required for this task (Ennaceur and Aggleton 1997; Barker and Warburton 2011b), the intact performance of the rats with MD and with mPFC lesions is consistent with a growing number of studies that report a lack of spatial memory deficit in rats following MD (Neave et al. 1993; Hunt and Aggleton 1998; Mitchell and Dalrymple-Alford 2005; Dolleman-van der Weel et al. 2009) or mPFC lesions (Barker et al. 2007).

Three animals in the mPFC group had a small amount of damage in the medial and ventral orbital cortex. Inspection of their individual discrimination ratios revealed that the pattern of performance of these animals was no different to that of the group as a whole. Thus these animals showed no deficits in the object recognition or object location tasks, but, consistent with the remainder of the mPFC lesion group, they showed poorer discrimination in the object-in-place and temporal order tasks. While with a small group (*n* = 3) one cannot draw firm conclusions, it does not appear from the pattern of performance in these animals that damage to the orbital frontal cortex contributes to the mnemonic deficits seen.

The demonstration that bilateral lesions in MD have no effect on an object location task allows us to exclude the possibility that the observed deficits in object-in-place and recency memory may be the result of the slight damage to the DG that occurred in some of the animals (unilateral damage in three cases, bilateral damage in one case). Further, as MD lesions had no effect on single-item recognition or object location, the object-in-place and recency recognition memory deficits are not the result of impaired object identification or object familiarity discrimination. As the object-in-place task requires the subject to encode four different objects it may be considered to be a more difficult task than the object recognition task. Thus in the present study the sample time in the object-in-place task is greater than that in the object recognition task to enable the subjects to spend longer exploring and encoding the objects. If we consider the performance of the sham group in both tasks it is clear that there is no difference in the levels of discrimination, indicating that the sham group does not find the object-in-place task more difficult. The absence of any differences in performance supports the conclusion that the impairments in object-in-place recognition memory following lesions in the MD and the mPFC reflect the importance of these brain regions selectively in associative recognition memory, and not the differences in task difficulty.

One previous study also found that lesions in the MD impaired a spontaneous recency memory task (Mitchell and Laiacona 1998), however the present study has extended upon this result by systematically exploring the role of the MD in recognition memory, and directly comparing this role with that of the mPFC for different types of information (single item, spatial location, object-in-place, and recency) using identical apparatus and stimulus types and similar retention delays. Further, the order in which the animals were tested in the battery of tasks interleaved the novel object recognition, object location, and object-in-place tasks, and thus it is unlikely that task order can account for the observed deficits. This study therefore demonstrates for the first time that the MD is selectively required for both associative and recency recognition memory in rats.

In light of these results, the Aggleton and Brown (1999) model of recognition memory, which placed the MD solely within a neural circuit for item familiarity through an interaction with the perirhinal cortex, requires a degree of reevaluation. In a recent review Aggleton et al. (2011) proposed a new model of thalamic contributions to recognition memory, the multi-effect multinuclei (MEMN) model. This model asserts that the MD can contribute to both familiarity and recollective processes either directly via an interaction with the prefrontal cortex or indirectly as a result of cortical diaschesis (Aggleton et al. 2011). This model is supported by the current findings regarding associative recognition, along with more recent clinical results (Pergola et al. 2012) that point to contributions from the parvicellular MD for recollective aspects of recognition. At the same time, there does appear to be a potential increase in the importance of the MD for familiarity-based object recognition in monkeys versus rats, potentially reflecting species differences in afferents to the MD (Russchen et al. 1987) and the greater influence of prefrontal areas for object recognition memory in primates (Aggleton et al. 2011).

To test the first of the possibilities raised by the MEMN model, i.e., that the involvement of the MD in recognition memory is through an interaction with the prefrontal cortex, we explored the functional importance of an interaction between these regions using a disconnection analysis. In animals with combined unilateral MD and mPFC lesions in opposite hemispheres (MD + mPFC Contra group), performance in the object-in-place and recency tasks was significantly impaired compared with that of animals with combined ipsilateral lesions (MD + mPFC Ipsi group). Here the MD + mPFC Ipsi group served as controls, as these animals sustained the same amount of damage, albeit in the same hemisphere, as animals in the MD + mPFC Contra group. While it is impossible to rule out entirely the possibility that the ipsilateral lesions produced some small effect on performance, the discrimination of the MD + mPFC Ipsi group in the object-in-place and recency tasks was significantly above chance. Therefore, damage to both regions within the same hemisphere appeared to spare performance, a finding consistent with numerous other studies that employ a disconnection technique to establish regional interdependencies (Floresco et al. 1997; Barker et al. 2007; Barker and Warburton 2011b). These results therefore suggest that the conjoint necessity for the MD and mPFC for successful recognition memory is due to the fact that these regions operate within an integrated neural circuit.

As the present study used permanent lesions to establish the importance of MD and mPFC in recognition memory, we cannot identify whether the contributions of the MD and mPFC are at the same or at different stages of recognition memory. We have previously shown that the mPFC is critical for both the acquisition and retrieval of object-in-place memory and that its role may be in the integration of object and spatial information or for the retrieval of object–spatial information through an engagement with the hippocampus (Barker et al. 2007; Barker and Warburton 2011b). Concerning the MD, one possibility is that this region acts as a critical relay between the medial temporal lobe and the mPFC, though such an explanation goes against present views of the role of thalamic nuclei (Sherman 2007; Tsanov et al. 2011) and leaves uncertain why the many direct connections linking temporal and frontal regions are not sufficient. It has also been suggested that ablation of the MD in primates produces hypoactivity in the mPFC (Parker and Gaffan 1998) and, therefore, the deficits in rats with MD lesions might be ascribed to such a frontal dysfunction. In the present study, histological analysis of the mPFC in animals with a bilateral MD lesion showed no indication of cell loss, yet previous studies have shown disruptive effects in regions distal to a lesion site, even when such regions appear normal by standard histological measures (Van Groen et al. 1993; Garden et al. 2009), and thus further study of potential diaschesis in the mPFC is required.

It is possible that during recognition memory performance the MD is critical for non-mnemonic cognitive processes such as behavioral flexibility (Block et al. 2007). If this were the case then during associative or recency recognition memory tasks, the MD–mPFC connection might be necessary to direct ongoing behavior toward, for example, the novel object–place configuration. Alternatively, the MD may be required for acquisition of new information or for retrieval. In primates the magnocellular MD has previously been shown to be necessary for acquisition but not for retrieval of preoperatively learnt object-in-place discriminations (Mitchell and Gaffan 2008). However, as such tasks are quite distinct from those used here, an examination of the separate roles of the rat MD in acquisition or retrieval is now warranted.

The present study adds to a growing body of evidence that for associative and recency recognition memory, information necessary for successful performance is held in multiple brain regions, including the perirhinal cortex, hippocampus, and MD. Further, it appears that each of these regions is required to interact with the mPFC (Hannesson et al. 2004; Barker et al. 2007; Barker and Warburton 2011b). Taken together these results show that during complex recognition memory tasks in which the processing of items requires a contextual (place or time) tag, the MD appears to have a key role within this neural network, Further, the results indicate that the nature of to-be-remembered determines which brain regions become engaged.

Recognition memory performance has been carefully examined in a limited number of clinical cases in which the MD is damaged, and results indicate an involvement of this thalamic nucleus in associative recognition (Aggleton et al. 2011; Pergola et al. 2012). Of particular interest is a study of a patient with an infarct involving the inferior capsular genu, which disrupted the connection between the thalamus (including the MD) and the prefrontal cortex (Schnider et al. 1996). On testing, nonassociative recognition memory was found to be normal while associative and temporal order recognition memory were both significantly impaired (Schnider et al. 1996). The precise locus of damage responsible for the memory deficits in this study is unclear as inevitably the lesion involved other regions, including the anterior thalamic nuclei and thalamic connections with the amygdala. However, the deficits in both temporal order and contextual recognition memory accord well with the results presented here.

In summary, results from this study demonstrate that the MD is critical for object-in-place and recency memory performance. Furthermore, for these recognition memory judgments, an interaction between the MD and the mPFC is equally necessary as the integrity of the structures themselves. Our previous studies revealed a neural circuit involving the hippocampus, perirhinal cortex, and mPFC for both associative recognition and recognition memory discriminations based on recency information (Barker et al. 2007; Barker and Warburton 2011a). The evidence now suggests that the MD is a further critical component of this circuit.

## Materials and Methods

### Subjects

Experiment 1 used 36 male rats and Experiment 2 used 24 male rats (DA strain; Bantin and Kingman, Hull, UK), weighing 220–290 g prior to testing. All the animals were housed in groups of four, under a 12-h light/dark cycle (light phase, 18.00 to 6.00) with ad libitum access to food and water. Behavioral testing was conducted during the dark phase of this cycle. All animal procedures were performed in accordance with UK Animals Scientific Procedures Act (1986) and associated guidelines. All efforts were made to minimize any suffering and the number of animals used.

### Surgery

#### Experiment 1

Rats received bilateral excitotoxic lesions in the mPFC or MD. Control animals received sham surgery; half the control animals received sham mPFC lesions, and the other half received sham MD surgeries. For the sham surgeries the animals underwent the same surgical procedures as the two lesion groups with the exception that no excitotoxin was injected once the needle had been lowered (*n* = 12 for all groups).

#### Experiment 2

All the rats received combined unilateral lesions in the MD and the mPFC. In one group these lesions were placed in the same hemisphere (MD + mPFC Ipsi group), while in the other group these lesions were placed in contralateral hemispheres (MD + mPFC Contra group) (*n* = 12 for both groups). The side of damage (i.e., left or right hemisphere), was counterbalanced within each group.

Before surgery all the rats were anesthetized (isoflurane: induction 4%; maintenance 2%–4%) and placed in a stereotaxic frame with the incisor bar set at +5 mm above the interaural line. The scalp was further anesthetized using lidocaine, cut, and retracted. After craniotomy, excitotoxic lesions to the target regions were made using *N-*methyl-D-aspartate (NMDA) dissolved in phosphate buffer (PB), injected through a 1-μL Hamilton syringe at the following coordinates relative to bregma: mPFC, anterior–posterior (AP) +2.7 mm, mediolateral (ML) ±0.7 mm, dorsoventral (DV) −4.5 mm and −2.2 mm, and AP +4.0 mm, ML ±0.7 mm, DV −3.5 mm and −2.0; MD, AP +3.7 mm, ML ±0.7 mm, DV +4.6. The coordinates for the mPFC lesion were calculated relative to bregma, while the coordinates for MD were calculated relative to ear-bar zero. For the mPFC lesion each NMDA injection (0.09 M, 0.28 μL) was made gradually over 4 min and the needle left in situ for a further 4 min. For the MD lesion, NMDA (0.12 M, 0.36 μL) was injected into each site gradually over 5 min and the needle left in situ for a further 5 min.

Once surgery was completed the skin was sutured and an antibiotic powder (Acramide) applied. All the animals received at least 5 mL of glucose saline subcutaneously and systemic analgesia intramuscularly (0.05 mL Vetergesic) before the end of surgery. Hypromellose eye drops were given at the beginning and end of surgery. The animals were allowed to recover for at least 10 d before habituation to the behavioral arena commenced.

### Histology

On completion of the behavioral tasks the animals were sacrificed by transcardial perfusion with PB followed by 4% paraformaldehyde (PFA). The brains were post-fixed in 4% PFA for a minimum of 24 h followed by 48 h in 30% sucrose in PB. Coronal sections (40 μm) were cut on a cryostat and the sections mounted directly onto gelatin-coated slides, stained using cresyl violet, and coverslipped using DPX mounting medium. Slides were then viewed under a light microscope and the extent of lesions recorded.

To assess the extent of the mPFC lesion all sections along the AP axis of each rat brain between +4.7 mm and +1.5 mm (relative to bregma) were selected and compared with those in a rat brain atlas (Swanson 1998). To assess the extent of the MD lesion, all sections along the AP axis of each rat brain between −1.5 and −4.20 (relative to bregma) were selected and compared with the rat brain atlas (Swanson 1998).

### Apparatus

Behavioral testing took place in a wooden open-topped arena (50-cm high, 95-cm wide, and 100-cm long), with gray walls and external black curtains to a height of 1.5 m to restrict distal cues. The floor was covered in sawdust, which served to conceal the identical green Duplo mats (15 cm × 15 cm), onto which the objects were attached to prevent them being displaced. In all the experiments the sawdust was cleaned between each animal. Exploration was monitored using an overhead camera and recorded onto videotape. The amount of object exploration was determined using in-house counting software on a computer within the room which, in response to a key press from the experimenter (one key for the left object, one key for the right object), recorded the amount of exploration completed within 20-sec time bins. Objects were constructed from Duplo and varied in color and size from 9 × 8 × 7 cm to 25 × 15 × 10 cm. New objects were used for every experiment.

### Behavioral procedures

#### Habituation

Prior to testing, all the animals were handled for a week and habituated to the empty arena for four days. For the first two of these days each cage of animals (four rats) was habituated to the arena together for 15 min. For the next two sessions, each rat was habituated individually for 5 min in the empty arena. Animals were also habituated individually for a 5-min period prior to the tasks in which the appearance of the arena had been altered from that of the standard arena (i.e., for the object location and object-in-place tasks).

#### Familiarity discrimination (a novel object recognition task)

In the sample phase, the animals were introduced to an arena containing two identical copies of one object. Animals were allowed to explore the two copies freely until they had completed a total of 40 sec of object exploration, or had spent 4 min in the arena. Animals were removed from the arena and placed in their home cage within the testing room, for a delay period (5 min or 3 h) during which time the arena and objects were cleaned. Following the delay, animals were placed back into the arena, which now contained another copy of the object seen in the sample phase and a novel object. The animal was allowed to explore the objects freely for 3 min and was then returned to its home cage. The object acting as the sample object and the position of the objects were counterbalanced across the animals. A representation of the procedure is shown in Figure 1A.

#### Spatial discrimination (object location task)

The object location task was conducted in a similar way to the object recognition task; however, two of the curtains surrounding the arena were removed to allow the rat to view extra-maze cues and one of the arena walls was painted a different color to provide an intra-maze cue. During the sample phase, animals were allowed to explore two identical copies of an object for 4 min before being removed from the arena for the delay period (5-min or 3-h delay in Experiment 1 and 3-h delay only in Experiment 2). Following the delay, the animals were placed back into the arena, which now contained two replicas of the objects from the sample phase. One object was replaced in the location previously occupied by a sample-phase object, but the other object was placed in a new location within the arena. Object exploration was recorded for 3 min. The position of the moved object was counterbalanced across rats. A representation of the procedure is shown in Figure 1B.

#### Associative recognition memory (object-in-place task)

Object-in-place testing was conducted in an arena identical to that used for the object location task. In this task, four different objects were placed in the four corners of the arena and during the sample phase the animals were allowed to explore these four objects for a 5-min sample phase. The animals were removed from the arena for the delay period (5 min or 3 h) and the objects cleaned with ethanol. In the test phase the positions of two of these objects were exchanged. The positions of the objects and particular objects moved were counterbalanced across animals. A representation of the procedure is shown in Figure 1C.

#### Recency recognition memory (recency task)

For this task the arena was identical to that used for the object recognition experiment. The task comprised two sample phases and one test trial. In the first sample phase, animals were allowed to explore two identical objects for 4 min before being returned to their home cage for a delay of 1 h. For the second sample phase, animals were placed back into the arena, which now contained two identical objects that were different to those seen in sample phase 1. Animals were allowed to explore these new objects for 4 min and then removed to their home cages. Following a 3-h delay, the animals were returned to the arena for the test phase where exploration of a copy of the object seen in sample phase 1 and a copy of the object from sample phase 2 was recorded. As the control animals in this task had relatively low baseline discrimination levels associated with appreciable variance, the task was repeated, with the left–right position of the objects from sample phase 1 and sample phase 2 counterbalanced and the data from the two runs were combined. Objects used in all sample phases and the locations of the objects were counterbalanced across the rats. A representation of the procedure is shown in Figure 1D.

### Behavioral measures and statistical analyses

Exploration of the objects, defined as the animal orientating its nose toward the object at a distance of <1 cm from the object, was measured with the experimenter blind to the lesion status of the animal. Any other behavior, such as looking around while sitting on or resting against the object or using the object to rear while looking around the arena, was not considered as exploration. Animals were excluded from the analysis on the basis of low exploration levels (<15 sec in the sample phase). A discrimination ratio was generated for each animal. The discrimination ratio was calculated as the time spent by each animal exploring the novel object minus that exploring the familiar object, divided by the total time spent exploring both objects. In the case of the object recognition task, the novel object was an object that had never been previously encountered, while for the object-in-place and object location tasks the novel stimulus was considered to be the object that had altered its position in the arena from that in the sample phase. In the case of the temporal order task, the ‘novel’ stimulus was the object encountered in the first sample phase. Group comparisons used ANOVA followed by post-hoc Newman–Keuls tests. Additional analysis examined whether individual groups had discriminated between the objects using a one-sample *t*-test (two-tailed) where significance was assumed when *P* < 0.05.

The recognition memory experiments were conducted in the following order: Object recognition with a 3-h delay, object location with a 5-min delay, object-in-place with a 5-min delay, object location with a 3-h delay, recency, object-in-place with a 3-h delay, and object recognition with a 5-min delay. The animals were run in this order to minimize the effects of task order on performance.
